# Boundary-aware dual-discriminator generative adversarial network for data augmentation in financial transaction fraud detection

**DOI:** 10.1371/journal.pone.0342095

**Published:** 2026-02-20

**Authors:** Honghao Zhu, Zhanchao Wang, Yu Xie, Jiamin Yao

**Affiliations:** 1 School of Computer and Information Engineering, Bengbu University, Bengbu, China; 2 College of Information Engineering, Shanghai Maritime University, Shanghai, China; University of Roehampton - Digby Stuart College, UNITED KINGDOM OF GREAT BRITAIN AND NORTHERN IRELAND

## Abstract

The rapid growth of digital payments exacerbates the challenges in Financial Transaction Fraud Detection (FTFD). These challenges stem primarily from an extreme class imbalance, where legitimate transactions greatly outnumber fraudulent ones. This imbalance significantly hampers the ability of FTFD models to accurately learn fraud patterns. Although existing data augmentation techniques have shown effectiveness in alleviating this problem, they are often negatively influenced by anomalous samples that diverge from the true fraud distribution due to fraudsters’ concealment strategies and the inherent complexity of fraudulent patterns. This divergence makes it challenging to accurately model the distribution of fraudulent activities. In this work, we propose a Boundary-Aware Dual-discriminator Generative Adversarial Network (BADGAN) to address the class imbalance issue in FTFD. BADGAN integrates a boundary sample classifier with a dual-constraint mechanism based on distance adversarial learning, allowing the generator to produce synthetic samples that both adhere to the distribution of real fraud data and maintain a distance from the decision boundary. This boundary-aware design emphasizes the optimization of sample quality near classification boundaries, thereby improving the downstream classifier’s ability to distinguish fraudulent behavior. Extensive experiments on both real-world and public datasets demonstrate that BADGAN outperforms its competitive peers in addressing the class imbalance issue, thereby enhancing the detection performance of FTFD models.

## 1 Introduction

The rapid proliferation of e-commerce and digital payment systems significantly transforms the landscape of global financial transactions. This transformation is further accelerated by the COVID-19 pandemic, solidifying online shopping as the dominant retail model. While these advancements substantially improve economic accessibility and consumer convenience, they also expand the attack surface, thereby enabling increasingly sophisticated financial fraud schemes [1,2]. The evolution of fraud tactics, combined with the increasing volume of digital financial activities, heightens the demand for robust Financial Transaction Fraud Detection (FTFD) systems. These systems must accurately identify fraudulent transactions while ensuring that legitimate financial flows remain uninterrupted and the user experience is not compromised. This presents a fundamental challenge, as traditional fraud detection methods, which are often reactive, struggle to keep pace with the evolving landscape of fraud.

Recent advancements in FTFD employ machine learning-based classifiers to differentiate between legitimate and fraudulent transactions [3]. While they demonstrate notable performance in controlled environments, their real-world deployment is limited by the inherent class imbalance in financial transaction datasets. Specifically, legitimate transactions overwhelmingly dominate the data, often accounting for more than 95% of all records, while fraudulent transactions represent only a small fraction [4]. This extreme class imbalance creates a significant bias in model training, causing classifiers to predominantly learn features associated with the majority class (legitimate transactions). As a result, the ability to detect the minority class (fraudulent transactions) is severely impaired, leading to poor generalization and an increased rate of false negatives [5]. Addressing this class imbalance constitutes a central focus of research in FTFD system development, with important implications for enhancing fraud detection accuracy and reliability in practical settings.

To mitigate the detrimental effects of class imbalance, various data augmentation techniques are proposed, including oversampling, undersampling, and synthetic data generation approaches [6,7]. Among these, Generative Adversarial Networks (GANs) [8] attract significant attention for their capacity to generate synthetic samples that replicate the distribution of fraudulent transactions and preserve data authenticity. GANs demonstrate substantial potential in capturing complex patterns within imbalanced datasets by modeling the underlying distribution of fraudulent activities. However, as fraud detection systems advance, fraudsters continuously adapt and refine their tactics. Modern fraudsters frequently exploit synthetic identities and adversarial machine learning techniques to obscure the decision boundaries between legitimate and fraudulent transactions [9,10]. This ongoing technological arms race exposes a fundamental vulnerability in conventional GAN-based augmentation methods. The same adversarial mechanisms that enable GANs to generate realistic samples also predispose them to incorporate deceptive patterns designed to mislead classifiers [11]. Consequently, GAN-based data augmentation techniques may fail to capture emerging fraud patterns or, more critically, inadvertently generate adversarial noise that compromises the performance of fraud detection models. The core challenge lies in GANs’ inability to reliably distinguish between authentic fraud patterns and adversarial perturbations, leading to suboptimal performance in detecting novel and sophisticated fraudulent activities.

To overcome these limitations and advance the state of the art in FTFD, we propose a novel Boundary-Aware Dual-discriminator Generative Adversarial Network (BADGAN) to address the class imbalance issue. Unlike traditional GANs, which may occasionally generate samples near decision boundaries, BADGAN alleviates this issue by incorporating boundary sample discriminators and boundary-distance constraints. These mechanisms ensure that the synthetic fraud samples not only replicate the distribution of authentically fraud patterns but also remain sufficiently distant from the decision boundaries, thereby reducing the risk of misclassification. By embedding these boundary-aware mechanisms, BADGAN mitigates the feature overlap commonly observed in GAN-based augmentation, enabling the generation of more representative synthetic data. This, in turn, enhances the ability of FTFD classifiers to accurately distinguish fraudulent transactions from legitimate ones, thereby improving detection performance in real-world scenarios.

The main contributions of this work to FTFD are summarized as follows:

1. It proposes a novel boundary-aware dual-discriminator generative adversarial network that generates synthetic fraud samples by authentically replicating fraud patterns while explicitly avoiding decision boundary regions. This approach addresses class imbalance and enhances the quality of training data for fraud detection classifiers.
2. It introduces a two-stage optimization strategy, where the real sample discriminator ensures conformity to fraud patterns, and the boundary sample discriminator enforces spatial separation from decision boundaries. This allows the generator to simultaneously learn fraud features and boundary integrity, producing synthetic samples that are both representative and discriminative.
3. It demonstrates the effectiveness of BADGAN through extensive experiments on real-world and public datasets. The results show that BADGAN enhances fraud detection performance by generating high-quality synthetic samples that effectively address class imbalance, thereby improving both detection accuracy and reliability in FTFD.

The remainder of this paper is structured as follows. Sect 2 reviews the related work. Sect 3 details the proposed methods. Sects 4 and 5 present the experimental setup and results, respectively. Sect 6 concludes this paper.

## 2 Related work

Class imbalance represents a pervasive challenge in machine learning, particularly in FTFD, where fraudulent transactions constitute only a small fraction of the overall dataset. This pronounced class imbalance induces systematic bias in learning algorithms, favoring the majority class (legitimate transactions) and impairing the model’s capacity to effectively detect fraudulent activities [12]. To mitigate this issue, existing methods are generally classified into two main strategies: data-level and model-level approaches [13].

### 2.1 Data-level methods

Data-level methods aim to address class imbalance by modifying the training dataset to improve its representation of the minority class [14]. These techniques generally include oversampling, undersampling, hybrid sampling, and generative modeling approaches [15].

Oversampling methods focus on increasing the representation of the minority class by generating synthetic samples. A widely adopted approach is the Synthetic Minority Oversampling Technique (SMOTE) [16], which generates synthetic samples by interpolating between existing minority instances and their nearest neighbors. Extensions of SMOTE, such as Borderline-SMOTE [17] and ADASYN [18], enhance sample diversity by concentrating on the ambiguous boundary regions or sparsely populated areas of the minority class. Despite their widespread adoption, these interpolation-based methods face notable limitations in the context of FTFD. Due to their reliance on interpolation, they often fail to capture the intricate and sometimes unique patterns inherent in fraudulent transactions. Additionally, these methods may generate synthetic samples in regions where fraudsters intentionally obfuscate or manipulate transaction features, thereby failing to reflect the true complexity of fraudulent behaviors [19]. Furthermore, these techniques are vulnerable to adversarial patterns crafted to exploit their interpolation logic, resulting in synthetic samples that fail to faithfully represent real-world fraud and consequently degrade the detection performance of FTFD systems [20].

Undersampling methods are designed to address class imbalance by reducing the number of majority class samples. Techniques such as Tomek Links (TL) [21] and Cluster Centroids (CC) [22] are commonly employed. TL removes overlapping instances near the decision boundary, while CC selects representative majority class samples based on clustering. While undersampling can effectively alleviate class imbalance, it risks discarding informative majority class instances that may contain critical patterns relevant to modeling fraudulent behaviors. Such a reduction in majority class samples may impair the model’s generalization ability and ultimately degrade fraud detection performance in real-world financial transaction settings [23].

Hybrid sampling methods integrate oversampling and undersampling techniques to construct more balanced and representative datasets. Representative approaches include Synthetic Minority Transfer Learning (SMTL) [24] and Synthetic Minority Enhanced Sampling (SMEN) [25], which enhance the representation of the minority class while preserving the overall data distribution. However, these approaches may fail to capture the complex, domain-specific characteristics of fraudulent transactions. Moreover, the excessive generation of synthetic samples can introduce noise into the dataset, further diminishing model generalizability and compromising the performance of fraud detection in practical FTFD applications [26].

### 2.2 Model-level methods

Model-level methods focus on enhancing a classifier’s ability to learn from imbalanced datasets without altering the underlying data distribution [27,28].

Cost-sensitive learning methods aim to address class imbalance by assigning higher penalties to the misclassification of fraudulent transactions, thus encouraging models to prioritize the detection of minority-class instances [29]. However, due to the dynamic nature of fraud, where fraudsters often mimic normal transaction patterns or adapt their strategies in response to evolving detection mechanisms [30], the static penalty structures inherent in cost-sensitive approaches are frequently inadequate. This limitation may lead to performance degradation over time, as these models struggle to accommodate the continuously evolving tactics employed by fraudsters [31].

Ensemble learning methods, such as Boosting (e.g., AdaBoost [32]) and Bagging (e.g., Random Forests [33]), improve model robustness by combining multiple classifiers. The former enhances sensitivity to rare fraud patterns by iteratively reweighting misclassified instances, while the latter reduces variance through the aggregation of predictions. Despite these advantages, ensemble methods remain vulnerable to manipulation. Fraudsters may exploit subtle behavioral variations or introduce noise to deceive base classifiers, thereby undermining the ensemble’s overall effectiveness [34,35]. Additionally, the substantial computational cost of ensemble methods limits their real-time applicability, a crucial consideration for practical fraud detection systems [36].

GANs gain increasing attention for augmenting minority class instances in fraud detection tasks. However, they often struggle with boundary discrimination [37]. The adversarial training process can lead to the generation of synthetic samples that cluster near decision boundaries or, more problematically, within dense regions of legitimate transactions. This limitation arises because the generator’s optimization objective emphasizes sample realism, often at the expense of preserving subtle yet critical discriminative features that differentiate fraudulent transactions from legitimate ones [38]. Enhanced architectures, such as Wasserstein GAN (WGAN) [39] and Roulette-wheel selection GAN (RGAN) [40], are proposed to address training instability. However, these advancements do not fundamentally resolve the issue of boundary confusion. Consequently, the synthetic samples generated by these models often exhibit feature distributions that overlap significantly with legitimate transactions, thereby misleading classifiers rather than improving their discriminative ability. This challenge persists because conventional GAN frameworks lack explicit mechanisms to enforce separation between class manifolds in the generated feature space.

Two categories of adversarial attacks are particularly prominent in recent AI security research: evasion attacks and model-poisoning attacks. Evasion attacks involve manipulating inputs during the inference stage to mislead the model’s predictions, whereas model-poisoning attacks intervene during training by injecting adversarial samples that alter the learned model parameters. Both attack types pose serious risks to the reliability and security of machine learning models, especially in sensitive domains such as fraud detection.

Therefore, the current data augmentation and model-level methods in FTFD exhibit inherent limitations in addressing the non-stationary nature of fraud distributions and the deliberate obfuscation of fraudulent patterns. A significant weakness lies in the inability of these methods to distinguish between genuine boundary ambiguity and fraudsters’ intentional attempts to obscure their activities, resulting in synthetic samples that oversimplify or distort the true characteristics of fraudulent transactions. Most traditional techniques assume a static distribution of fraud, overlooking the adaptive strategies employed by fraudsters to blur class boundaries and mimic legitimate behavior [41,42]. As a result, models trained on such synthetic data may perform well on historical fraud patterns but struggle to detect more sophisticated fraud schemes that exploit these weaknesses. To overcome these challenges, future approaches must not only preserve the underlying structure of fraudulent transactions but also explicitly counteract adversarial obfuscation during sample generation.

## 3 Proposed method

The increasing sophistication of fraudulent tactics highlights fundamental limitations in existing GAN-based augmentation methods. Their unimodal discrimination is insufficient to address the dual challenges of FTFD: preserving fidelity to authentic fraud distributions while effectively navigating regions of boundary ambiguity. To overcome these challenges, we propose BADGAN, an enhanced GAN framework that integrates a boundary sample discriminator and distance-adversarial learning, enabling the generator to produce high-quality samples that not only align with genuine fraud distributions but also maintain a significant distance from decision boundaries.

**Fig 1:**
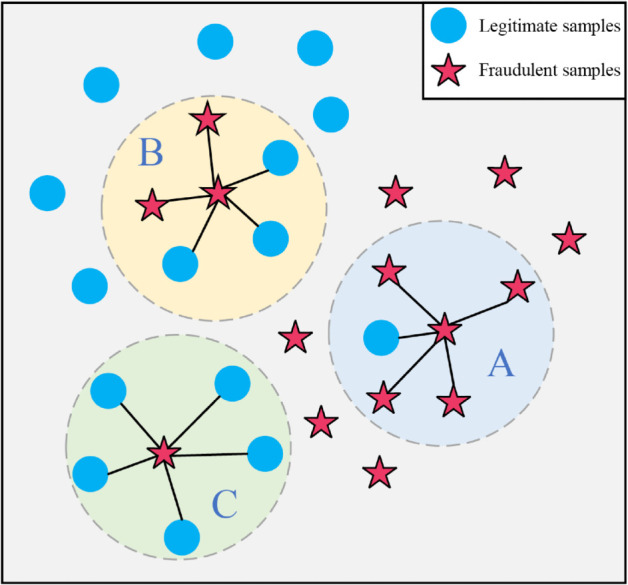
Boundary sample categorization using Borderline-SMOTE.

To enhance boundary discrimination, we incorporate Borderline-SMOTE [17] to identify and process boundary samples from the minority class, as illustrated in Fig 1. The choice of Borderline-SMOTE is motivated by its ability to focus specifically on instances that lie near the decision boundary, where fraudulent patterns are most prone to ambiguity. This targeted focus on boundary samples allows Borderline-SMOTE to generate more discriminative synthetic data, which is essential for capturing the subtle nuances of fraud. The minority class samples are categorized into three types:

- A (“safe”): Instances for which more than half of the nearest neighbors belong to the minority class, typically corresponding to genuine fraudulent transaction patterns.
- B (“dangerous”): Instances that are predominantly surrounded by majority-class neighbors, often reflecting fraudsters’ attempts to obscure fraudulent behavior and thereby increasing the likelihood of classifier confusion.
- C (“noise”): Instances that are entirely surrounded by majority-class samples, generally indicating anomalies arising from data collection or labeling processes.

In our preprocessing pipeline, Borderline-SMOTE is employed to strategically generate synthetic boundary samples by interpolating around high-risk instances. It focuses on the critical boundary region, where fraudulent patterns are most susceptible to ambiguity. By generating synthetic samples in these regions, Borderline-SMOTE effectively expands the coverage of boundary characteristics while preserving their essential features. These synthesized samples are subsequently used as specialized training data for the boundary sample classifier and are labeled as boundary fraudulent samples, thereby explicitly reflecting their role in enhancing boundary discrimination.

**Fig 2:**
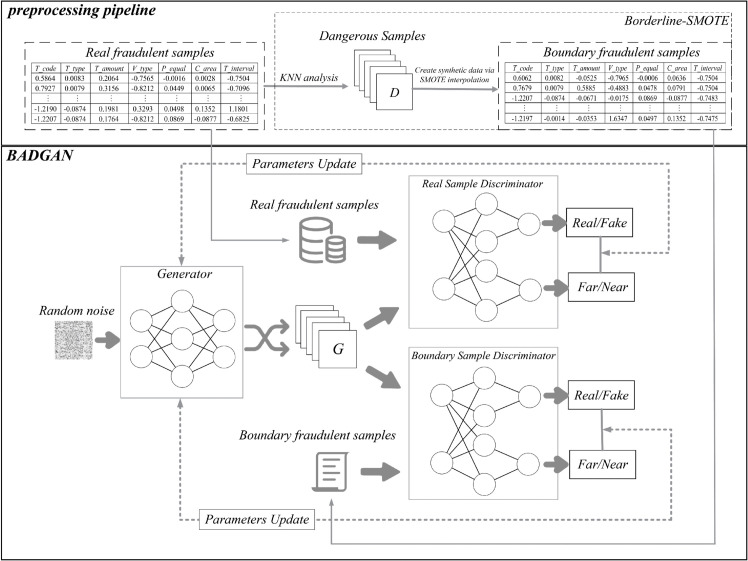
Illustration of BADGAN.

As shown in Fig 2, BADGAN employs a dual-discriminator architecture, with each discriminator performing a distinct yet complementary function. The real sample discriminator *D*_*r*_ is trained on both genuine fraudulent samples and generator outputs, guiding the generator to produce synthetic samples that not only resemble authentic fraudulent behavior but also capture the intricate patterns characteristic of fraud. This ensures that the generated samples align closely with the true nature of fraudulent transactions. Simultaneously, the boundary sample discriminator *D*_*b*_ operates on boundary fraudulent samples—instances that are particularly prone to misclassification due to their proximity to the decision boundary—along with generator outputs. *D*_*b*_ plays a critical role in penalizing the generator when synthetic samples overly align with boundary features, thereby preventing the generation of ambiguous samples. Instead, it encourages the production of samples with clear and identifiable fraudulent traits, which improves both the authenticity of the synthetic data and the clarity of classification boundaries.

This dual-discriminator framework ensures that BADGAN generates synthetic samples that both improve the overall quality of fraud detection and reduce the impact of boundary ambiguity. By focusing on boundary regions, we mitigate the risk of generating misleading samples that could hinder classifier performance. Furthermore, this approach enhances the robustness of the generated fraud patterns, making them more suitable for downstream fraud detection tasks. In doing so, BADGAN effectively balances fidelity to real-world fraud distributions with the avoidance of regions where fraud patterns overlap with legitimate data, thus optimizing the model’s overall FTFD performance.

The adversarial optimization objectives for *D*_*r*_ and *D*_*b*_ are defined as follows:

$${ℒ}_{r}^{adv}={𝔼}_{x\sim {𝒫}_{f}}[\log D_{r}^{adv}(x_{f})]+{𝔼}_{z\sim {𝒫}_{z}}[\log(1-D_{r}^{adv}(G(z)))],$$ (1)

$${ℒ}_{b}^{adv}={𝔼}_{x\sim {𝒫}_{b}}[\log D_{b}^{adv}(x_{b})]+{𝔼}_{z\sim {𝒫}_{z}}[\log(1-D_{b}^{adv}(G(z)))],$$ (2)

where *x*_*f*_, *x*_*b*_, and *z* denote real fraudulent samples, boundary fraudulent samples, and random noise, respectively. $D_{r}^{adv}(G(z))$ and $D_{r}^{adv}(x_{f})$ respectively denote the probabilities assigned to synthetic and real fraudulent samples, ranging from 0 to 1. *D*_*b*_ similarly aims to distinguish synthetic samples *G*(*z*) from boundary samples *x*_*b*_. During training, both discriminators strive to maximize their respective objectives by increasing values $D_{r}^{adv}(x_{f})$ and $D_{b}^{adv}(x_{b})$ while reducing $D_{r}^{adv}(G(z))$ and $D_{b}^{adv}(G(z))$.

Inspired by the adversarial training paradigm of GANs, the generator’s optimization objective is designed to synthesize samples that successfully deceive the fraud discriminator *D*_*r*_ while preserving the critical fraudulent characteristics. Additionally, the generator is explicitly guided to avoid producing samples that resemble boundary patterns, further ensuring that synthetic transactions are not misclassified as legitimate. This comprehensive adversarial optimization objective ensures that the generator produces high-quality, realistic fraudulent data that is useful for training robust fraud detection models.

$${ℒ}_{g}^{adv}={𝔼}_{z\sim {𝒫}_{z}}[\log(1-D_{r}^{adv}(G(z)))]+{𝔼}_{z\sim {𝒫}_{z}}[\log D_{b}^{adv}(G(z))].$$ (3)

To further enhance the discriminative capacity of the dual-discriminator framework, we introduce a distance-adversarial learning mechanism that explicitly improves the separation between fraudulent transactions and boundary fraudulent samples in the feature space. This is achieved by designing specific loss functions that guide the discriminators in distinguishing between clearly identifiable fraudulent transactions and those at risk of misclassification owing to their proximity to the decision boundary. In addition to outputting adversarial probabilities $D_{r}^{adv}$ and $D_{b}^{adv}$, the discriminators *D*_*r*_ and *D*_*b*_ also provide boundary-proximity scores, denoted as $D_{r}^{dist},D_{b}^{dist}\in (0,1)$. A value closer to 1 indicates that the input sample is considered farther from the decision boundary, while a value approaching 0 suggests that the sample is near the boundary. The distance optimization objectives for *D*_*r*_ and *D*_*b*_ are formulated as follows:

$${ℒ}_{r}^{dist}={𝔼}_{x\sim {𝒫}_{f}}[\log D_{r}^{dist}(x_{f})]+{𝔼}_{z\sim {𝒫}_{z}}[\log D_{r}^{dist}(G(z))],$$ (4)

$${ℒ}_{b}^{dist}={𝔼}_{x\sim {𝒫}_{b}}[\log(1-D_{b}^{dist}(x_{b}))]+{𝔼}_{z\sim {𝒫}_{z}}[\log D_{b}^{dist}(G(z))],$$ (5)

For boundary samples, the objective is for $D_{b}^{dist}(x)\to 0$. To achieve this, we employ the loss function $\log(1\;-\;D_{b}^{dist}(x))$, which drives the distance score of boundary samples toward zero, thereby encouraging them to remain close to the decision boundary. Conversely, for non-boundary samples, we aim to enforce $D_{b}^{dist}(G(z)),D_{r}^{dist}(x_{f}),$ and $D_{r}^{dist}(G(z))\to 1$, using the loss function $\log D_{b}^{dist}(G(z))$ to encourage these samples to be positioned farther from the decision boundary. This design encourages the generator to synthesize samples with well-separated class characteristics, thereby minimizing the influence of ambiguous boundary samples on the training process.

$${ℒ}_{g}^{dist}={𝔼}_{z\sim {𝒫}_{z}}[\log D_{r}^{dist}(G(z))]+{𝔼}_{z\sim {𝒫}_{z}}[\log D_{b}^{dist}(G(z))].$$ (6)

By incorporating the distance loss, the generated samples are explicitly encouraged to move away from the decision boundary, reducing the likelihood of misclassification in ambiguous regions. The overall optimization objectives for *D*_*r*_, *D*_*b*_, and *G* are defined as follows:

$${ℒ}_{r}={ℒ}_{r}^{adv}+{ℒ}_{r}^{dist},$$ (7)

$${ℒ}_{b}={ℒ}_{b}^{adv}+{ℒ}_{b}^{dist},$$ (8)

$${ℒ}_{g}={ℒ}_{g}^{adv}+{ℒ}_{g}^{dist}.$$ (9)

As shown in Fig 2, both *D*_*r*_ and *D*_*b*_ output probabilities in the range of 0 to 1 and are jointly optimized together with the generator. BADGAN improves the quality of generated samples and their compatibility with downstream classifiers by introducing a boundary-aware mechanism. Specifically, *D*_*b*_ models the decision boundary region explicitly, compelling the generator to produce fraudulent samples that avoid ambiguous boundary regions. The distance loss further ensures that the generated samples align with the underlying distribution of real fraudulent transactions in the feature space.

This dual optimization framework ensures that the generated samples preserve the dynamic characteristics of genuine fraud—such as sudden high-frequency transactions and abnormal fluctuations in transaction amounts—while simultaneously enabling higher classification confidence. As a result, the proposed augmentation method effectively mitigates class imbalance in FTFD, enabling downstream models to achieve superior detection performance even in low-supervision settings. The training process for BADGAN is outlined in Algorithm 1. The time complexity of the algorithm is $O(n_{e}\cdot k\cdot n_{b}\cdot d)$, where *n*_*e*_, *k*, *n*_*b*_, and *d* denote the number of training epochs, iterations per epoch, batch size, and features per sample, respectively.

**Algorithm 1 Training algorithm of BADGAN.**

**Input:** Fraudulent samples distribution ${𝒫}_{f}$, Boundary samples distribution ${𝒫}_{b}$, Noise distribution ${𝒫}_{z}$, Generator *G* with parameters $\theta_{G}$, Discriminators *D*_*r*_, *D*_*b*_ with parameters $\theta_{D_{r}}$, $\theta_{D_{b}}$, Training epochs *n*_*e*_, batch size *n*_*b*_

**Output:** Trained BADGAN model $\left(G,D_{r},D_{b}\right)$

1: Initialize *G*, *D*_*r*_, *D*_*b*_

2: **for** *epoch* = 1 **to** *n*_*e*_ **do**

3:   **for** *k* steps **do**

4:    Sample batch $\{x_{i}{\}}_{i=1}^{n_{b}}\sim {𝒫}_{f}$ {Fraudulent samples}

5:    Sample batch $\{x_{i}^{b}{\}}_{i=1}^{n_{b}}\sim {𝒫}_{b}$ {Boundary samples}

6:    Sample batch $\{z_{i}{\}}_{i=1}^{n_{b}}\sim {𝒫}_{z}$ {Noise vectors}

7:    **Update *D***_***r***_ **by ascending its stochastic gradient:**

8:    $\nabla_{\theta_{D_{r}}}\frac{1}{n_{b}}\sum_{i=1}^{n_{b}}\left[\log D_{r}^{adv}(x_{i})+\log(1-D_{r}^{adv}(G(z_{i})))\right]$

9:    $\nabla_{\theta_{D_{r}}}\frac{1}{n_{b}}\sum_{i=1}^{n_{b}}\left[\log D_{r}^{dist}(x_{i})+\log D_{r}^{dist}(G(z_{i}))\right]$

10:    **Update *D***_***b***_ **by ascending its stochastic gradient:**

11:    $\nabla_{\theta_{D_{b}}}\frac{1}{n_{b}}\sum_{i=1}^{n_{b}}\left[\log D_{b}^{adv}(x_{i}^{b})+\log(1-D_{b}^{adv}(G(z_{i})))\right]$

12:    $\nabla_{\theta_{D_{b}}}\frac{1}{n_{b}}\sum_{i=1}^{n_{b}}\left[\log(1-D_{b}^{dist}(x_{i}^{b}))+\log D_{b}^{dist}(G(z_{i}))\right]$

13:   **end for**

14:   Sample batch $\{z_{i}{\}}_{i=1}^{n_{b}}\sim {𝒫}_{z}$

15:   **Update *G* by ascending its stochastic gradient:**

16:   $\nabla_{\theta_{G}}\frac{1}{n_{b}}\sum_{i=1}^{n_{b}}\left[\log(1-D_{r}^{adv}(G(z_{i})))+\log D_{b}^{adv}(G(z_{i}))\right]$

17:   $\nabla_{\theta_{G}}\frac{1}{n_{b}}\sum_{i=1}^{n_{b}}\left[\log D_{r}^{dist}(G(z_{i}))+\log D_{b}^{dist}(G(z_{i}))\right]$

18: **end for**

19: **Return** $\left(G,D_{r},D_{b}\right)$

## 4 Experimental setup

### 4.1 Datasets

We obtain a comprehensive transaction dataset from a leading Chinese financial institution, comprising 5.12 million records involving 107,192 distinct clients. The dataset exhibits a significant class imbalance, with fraudulent transactions (labeled as “1”) representing only a small fraction relative to legitimate transactions (labeled as “0”). To ensure a robust evaluation of model performance, we employ a time-based partitioning strategy that preserves chronological order. Specifically, transactions from January are used for training, while transactions from February serve as the first test set. This partitioning scheme is extended to create six consecutive monthly evaluation phases, concluding on June. As summarized in Table 1, this temporal partitioning preserves the evolution of transaction patterns and prevents data leakage between training and testing phases, thereby ensuring the integrity of the evaluation process.

**Table 1:** Data partitioning.

Dataset	#Legitimate	#Fraudulent	#Total	#Ratio
Jan.	656416	28175	684591	0.043
Feb.	657899	33762	691661	0.051
Mar.	226206	10601	236807	0.047
Apr.	1229764	23271	1253035	0.019
May.	1189117	27122	1216239	0.023
Jun.	1017816	24898	1042714	0.024

### 4.2 Benchmarks

To comprehensively evaluate the performance of BADGAN, we benchmark it against ten competitive peers for addressing class imbalance. This allows us to rigorously assess the effectiveness, strengths, and limitations of BADGAN across various datasets and evaluation metrics. Our comparison not only validates the model’s capability in handling class imbalance but also highlights its relative advantages over existing solutions in fraud detection.

1. SMOTE [16] synthesizes new minority class samples by interpolating between existing instances and their nearest neighbors. This enhances sample diversity and supports more robust classifier decision boundaries.
2. SMOTE with Edited Nearest Neighbors (SMEN) [25] combines SMOTE’s oversampling technique with Edited Nearest Neighbors (ENN) data refinement. This approach generates synthetic samples while removing mislabeled or ambiguous majority instances to improve data quality.
3. SMOTE with Tomek Links (SMTL) [24] first applies SMOTE to enrich the minority class representation, followed by Tomek Links to eliminate closely spaced majority class instances. This combination sharpens class separation and enhances dataset structure.
4. ADASYN [18] generates synthetic samples adaptively, focusing on harder-to-learn minority instances. By leveraging local data density, ADASYN tailors sample creation to areas where classifiers need the most support.
5. Cluster Centroids (CC) [22] clusters majority class samples and retains the centroids of these clusters as representative samples. This reduces the number of majority class samples while preserving the overall data distribution and minimizing loss of minority information.
6. Tomek Links (TL) [21] identifies and removes pairs of samples that are close neighbors but belong to different classes. This process reduces class overlap and enhances boundary clarity, aiding in the construction of more discriminative models.
7. VGAN [8] introduces a generator-discriminator framework for data synthesis. By iteratively refining the generator’s output through adversarial feedback, the model generates realistic synthetic samples that reflect the true data distribution.
8. Semi-supervised GAN (SGAN) [43] enhances fraud detection by combining semi-supervised adversarial training with an anomaly density-guided selection process. SGAN targets high-risk fraudulent patterns and incorporates a behavioral deviation penalty to minimize overlap with legitimate transactions, generating more discriminative synthetic fraud samples for model training.
9. Roulette-Wheel-Selection-based GAN (RGAN) [40] published in 2023 introduces a targeted sampling strategy to emphasize regions of inter-class overlap, guiding the generator to capture finer fraud characteristics and improve the quality of synthetic minority data near decision boundaries.
10. Density-based Wasserstein Generative Adversarial Network (DWGAN) [44] published in 2025 enhances fraud sample generation by combining Wasserstein adversarial training with density-guided sample selection. This method emphasizes representative fraudulent regions and incorporates penalties to reduce overlap with legitimate behaviors, producing more distinctive and high-quality synthetic samples.

To streamline subsequent comparisons, we adopt the following notations: SMEN, SMTL, ADASYN, SMOTE, TL, CC, SGAN, RGAN, DWGAN, VGAN, and BADGAN are denoted as *M*_1_, *M*_2_, *M*_3_, *M*_4_, *M*_5_, *M*_6_, *M*_7_, *M*_8_, *M*_9_, *M*_10_, and *M*_11_, respectively. These abbreviations are applied consistently across all tables, figures, and discussions to enhance readability while ensuring methodological clarity.

### 4.3 Evaluation criteria

To evaluate the performance of BADGAN in FTFD, we utilize two widely adopted metrics: F1-Score (*F*_1_) and Geometric Mean (*G*_*m*_). These metrics are particularly suitable for binary classification problems, such as fraud detection, where the minority class—fraudulent transactions—requires special attention due to its significant underrepresentation [28,45]. Both metrics are derived from the confusion matrix, as shown in Table 2, which quantifies a classifier’s predictions against the ground truth. In FTFD, the confusion matrix consists of four components:

**Table 2:** Confusion matrix of FTFD.

	Actual fraudulent	Actual legitimate
Predicted fraudulent	*T*_*P*_	*F*_*P*_
Predicted legitimate	*F*_*N*_	*T*_*N*_

- True Positive (*T*_*P*_): The number of fraudulent transactions correctly identified as fraud.
- False Positive (*F*_*P*_): The number of legitimate transactions incorrectly classified as fraud.
- False Negative (*F*_*N*_): The number of fraudulent transactions incorrectly classified as legitimate.
- True Negative (*T*_*N*_): The number of legitimate transactions correctly identified as non-fraudulent.

*F*_1_ balances precision and recall, serving as a critical metric in FTFD. It ensures that the model effectively identifies fraudulent transactions (high recall) while maintaining a low rate of false positives (high precision). This balance is crucial for reliable fraud detection systems in financial contexts [46].

$$F_{1}=\frac{2\times T_{P}}{2\times T_{P}+F_{P}+F_{N}}.$$ (10)

*G*_*m*_ evaluates classification balance by combining the true positive rate (sensitivity) and the true negative rate (specificity). This metric is particularly valuable in the context of imbalanced datasets, as it ensures robust performance on both the minority (fraudulent) and majority (legitimate) classes, thereby mitigating bias towards either class [47].

$$G_{m}=\sqrt{\frac{T_{p}}{T_{p}+F_{N}}\times \frac{T_{N}}{T_{N}+F_{P}}}.$$ (11)

By jointly considering *F*_1_ and *G*_*m*_, we obtain a more comprehensive assessment of the model’s discriminative capability. This joint evaluation emphasizes both sensitivity to fraudulent cases and robustness in identifying legitimate transactions. Together, these metrics provide a robust and informative evaluation of synthetic data quality and its impact on downstream FTFD models.

### 4.4 Parameter settings

For all experiments, the nearest-neighbor parameter in Borderline-SMOTE is set to *k* = 5, with its effectiveness verified through sensitivity analysis in Sect 5.3. The BADGAN model is trained using a batch size of 64 for 1000 epochs, striking a balance between capturing underlying patterns and mitigating the risk of overfitting. To maintain equilibrium between the generator and the two discriminators, each round of discriminator training is followed by an additional round of generator training. This approach prevents the generator from being overly suppressed by dominant discriminators. Both the generator and discriminators are optimized using the Adam optimizer [48] with a learning rate of 0.0001, which enhances training stability and reduces the risk of mode collapse [49].

For simplicity, and to minimize the influence of network complexity on performance evaluation, the generator is implemented as a two-layer fully connected neural network. Adversarial training employs Binary Cross-Entropy (BCE) loss, which provides well-defined optimization objectives, stabilizes gradients, and maintains stable adversarial dynamics between the generator and the discriminators, thereby improving both training efficiency and overall model performance [50,51].

For the remaining sampling methods, key hyperparameters (e.g., neighborhood size in SMOTE-based approaches) are configured according to their original implementations or standard practices in imbalanced learning. GAN-based models adopt network architectures and optimization settings consistent with those of BADGAN where applicable, to ensure a fair comparison while preserving their respective architectural design principles and maintaining optimal performance [52]. All methods are fine-tuned following established benchmarks or default recommendations in the class imbalance literature.

## 5 Experimental results and analysis

### 5.1 Experiments on FTFD

To assess BADGAN’s ability to address class imbalance in FTFD, we conduct extensive testing on real-world financial transaction datasets spanning multiple time periods. The model’s performance is benchmarked against ten state-of-the-art approaches, using three standard classifiers: Support Vector Classifier (SVC), Logistic Regression (LR), and Multilayer Perceptron (MLP). For a fair comparison, all GAN-based competitors utilize consistent generator and discriminator architectures. Experimental results are based on the mean values of ten repeated trials, ensuring statistical reliability. For brevity, the average value and ranking are denoted by $A_{v}$ and *A*_*r*_, respectively, in the subsequent results.

Training efficiency and inference speed (the average time of training the model and detecting transactions) are shown at the bottom of Tables 3, 4, and 5. However, despite the increase in model complexity due to the adoption of the dual-classifier structure, the time for both training and inference remains at an acceptable level, ensuring that the model’s high performance does not come at the cost of reduced efficiency.

**Table 3:** FTFD performance of eleven methods When SVC is used as a base classifier.

Train	Test	Criteria	$M_{1}$	$M_{2}$	$M_{3}$	$M_{4}$	$M_{5}$	$M_{6}$	$M_{7}$	$M_{8}$	$M_{9}$	$M_{10}$	$M_{11}$
Jan.	Feb.	*F*_1_	0.3755	0.3360	0.3333	0.3814	0.4606	0.4421	0.4634	0.4721	0.4594	0.4830	**0.5118**
		*G*_*m*_	0.7566	0.7080	0.7517	0.7584	0.7763	0.7844	0.7671	0.8028	0.7760	0.8182	**0.8406**
Feb.	Mar.	*F*_1_	0.8899	0.8951	0.6126	0.8888	0.5676	0.5993	0.8210	0.7035	0.9458	0.7408	**0.9650**
		*G*_*m*_	0.9857	0.9864	0.9298	0.9856	0.7301	0.8323	0.9755	0.8998	0.9921	0.9657	**0.9965**
Mar.	Apr.	*F*_1_	**0.8895**	0.8828	0.8863	0.8721	0.8638	0.8774	0.8876	0.8493	0.8655	0.8815	0.8879
		*G*_*m*_	0.8960	0.8910	0.8931	0.8852	**0.8988**	0.8923	0.8932	0.8962	0.8925	0.8909	0.8946
Apr.	May.	*F*_1_	0.7344	0.6816	0.5755	0.6684	0.6144	0.6342	0.5645	0.7445	0.7175	0.5784	**0.8049**
		*G*_*m*_	0.9102	0.9328	0.8991	0.9301	**0.9538**	0.9353	0.7268	0.9175	0.9156	0.7776	0.9488
May.	Jun.	*F*_1_	0.5709	0.5700	0.4422	0.5665	0.6564	0.6223	0.7414	0.6274	0.7174	0.6233	**0.7863**
		*G*_*m*_	0.9071	0.9069	0.8619	0.9058	0.9026	0.9198	0.8729	0.9081	0.8783	0.9201	**0.9291**
$A_{v}$		*F*_1_	0.6920	0.6731	0.5700	0.6754	0.6326	0.6351	0.6956	0.6794	0.7411	0.6614	**0.7912**
		*G*_*m*_	0.8911	0.8850	0.8671	0.8930	0.8523	0.8728	0.8471	0.8849	0.8909	0.8745	**0.9219**
*A*_*r*_		*F*_1_	5.0000	6.4000	9.0000	7.4000	7.6000	7.6000	5.2000	5.8000	4.8000	6.0000	**1.2000**
		*G*_*m*_	5.8000	6.6000	8.8000	7.2000	5.2000	5.6000	7.8000	4.8000	6.2000	6.2000	**1.8000**
Training efficiency(ms)			54.98	57.03	22.89	50.00	79.44	4.99	24.00	58.60	84.00	61.59	31.99
inference speed(ms)			18.01	17.52	6.07	14.59	42.02	6.98	6.96	14.98	20.92	17.28	9.97

**Table 4:** FTFD performance of eleven methods when LR is used as a base classifier.

Train	Test	Criteria	$M_{1}$	$M_{2}$	$M_{3}$	$M_{4}$	$M_{5}$	$M_{6}$	$M_{7}$	$M_{8}$	$M_{9}$	$M_{10}$	$M_{11}$
Jan.	Feb.	*F*_1_	0.3093	0.3028	0.2503	0.3485	0.4441	0.3750	0.4061	0.4004	0.4431	0.3716	**0.4603**
		*G*_*m*_	0.7519	0.7441	0.6592	0.7256	0.8195	0.8092	0.8035	0.8051	0.8191	0.8083	**0.8257**
Feb.	Mar.	*F*_1_	0.8642	0.8632	0.7339	0.8919	0.9510	0.8218	0.8046	0.9266	0.9468	0.8432	**0.9624**
		*G*_*m*_	0.9803	0.9801	0.9603	0.9860	0.9869	0.9756	0.9709	0.9843	0.9911	0.9686	**0.9939**
Mar.	Apr.	*F*_1_	0.8686	0.8678	0.8755	**0.8879**	0.8372	0.8710	0.8377	0.8645	0.8403	0.8661	0.8771
		*G*_*m*_	0.8832	0.8836	0.8895	**0.8946**	0.8555	0.8865	0.8569	0.8807	0.8889	0.8821	0.8910
Apr.	May.	*F*_1_	0.5951	0.5918	0.6363	0.6460	0.8830	0.8426	0.6431	0.8885	0.8909	0.8824	**0.9017**
		*G*_*m*_	0.9268	0.9232	0.8933	0.9263	0.9284	**0.9290**	0.8759	0.9277	0.9279	0.9070	**0.9290**
May.	Jun.	*F*_1_	0.8296	0.8269	0.7133	0.5696	0.8618	0.7759	0.7420	0.8673	0.9029	0.8551	**0.9307**
		*G*_*m*_	**0.9679**	0.9675	0.9513	0.9067	0.9607	0.9589	0.9427	0.9590	0.9291	0.9644	0.9329
$A_{v}$		*F*_1_	0.6934	0.6905	0.6419	0.6688	0.7954	0.7373	0.6867	0.7895	0.8048	0.7637	**0.8264**
		*G*_*m*_	0.9020	0.8997	0.8707	0.8878	0.9102	0.9118	0.8900	0.9114	0.9112	0.9061	**0.9145**
*A*_*r*_		*F*_1_	7.2000	8.2000	8.8000	6.4000	4.6000	6.6000	8.2000	4.6000	3.8000	6.4000	**1.2000**
		*G*_*m*_	5.6000	6.4000	8.4000	6.6000	4.6000	4.8000	9.0000	6.0000	4.6000	7.0000	**2.8000**
Training efficiency(ms)			9.99	6.40	8.16	10.99	6.02	4.09	24.20	14.13	18.97	14.95	16.00
inference speed(ms)			1.09	0.97	1.55	1.00	0.89	0.96	1.13	0.86	1.20	1.12	0.98

**Table 5:** FTFD performance of eleven methods when MLP is used as a base classifier.

Train	Test	Criteria	$M_{1}$	$M_{2}$	$M_{3}$	$M_{4}$	$M_{5}$	$M_{6}$	$M_{7}$	$M_{8}$	$M_{9}$	$M_{10}$	$M_{11}$
Jan.	Feb.	*F*_1_	0.2696	0.2627	0.3069	0.3298	0.3467	0.3073	0.3617	0.3555	0.3455	0.3128	**0.3699**
		*G*_*m*_	0.6966	0.6847	0.7491	0.7005	0.7828	0.7488	0.7794	0.7886	0.7818	0.7516	**0.7991**
Feb.	Mar.	*F*_1_	0.9278	0.9720	0.9576	0.8961	0.9745	0.8876	0.9770	0.9001	0.9782	0.8371	**0.9807**
		*G*_*m*_	0.9774	0.9925	0.9805	0.9865	0.9939	0.9843	**0.9954**	0.9858	0.9943	0.9778	0.9946
Mar.	Apr.	*F*_1_	0.8356	0.8324	0.8367	0.8088	0.7943	0.8372	0.8028	0.8128	0.8340	0.8292	**0.8762**
		*G*_*m*_	0.8845	0.8816	0.8846	0.8619	0.8475	**0.8860**	0.8550	0.8640	0.8831	0.8787	0.8830
Apr.	May.	*F*_1_	0.8286	0.8267	0.8068	0.6290	0.8377	0.8656	0.7478	0.8394	0.8085	0.7845	**0.8927**
		*G*_*m*_	0.8479	0.8394	0.8222	0.9244	0.8569	0.9254	0.8212	0.8584	0.8556	0.8519	**0.9256**
May.	Jun.	*F*_1_	0.9457	0.9300	0.9278	0.5694	0.9309	0.7336	0.9355	0.9327	0.9324	0.9428	**0.9528**
		*G*_*m*_	0.9505	0.9366	0.9389	0.9058	0.9342	0.9540	0.9396	0.9381	0.9356	0.9575	**0.9693**
$A_{v}$		*F*_1_	0.7615	0.7648	0.7672	0.6466	0.7768	0.7263	0.7650	0.7681	0.7797	0.7413	**0.8145**
		*G*_*m*_	0.8714	0.8670	0.8751	0.8758	0.8831	0.8997	0.8781	0.8870	0.8901	0.8835	**0.9143**
*A*_*r*_		*F*_1_	5.6000	7.2000	7.0000	9.2000	6.0000	6.4000	5.8000	5.4000	5.0000	7.4000	**1.0000**
		*G*_*m*_	7.2000	7.8000	6.8000	7.6000	6.6000	4.4000	6.4000	5.6000	5.2000	6.4000	**2.0000**
Training efficiency(ms)			845.18	872.98	940.77	973.39	442.42	646.98	917.73	860.47	1094.16	912.37	907.32
inference speed(ms)			4.12	3.00	3.13	3.02	3.24	5.64	2.01	2.95	3.05	2.03	2.97

**Fig 3:**
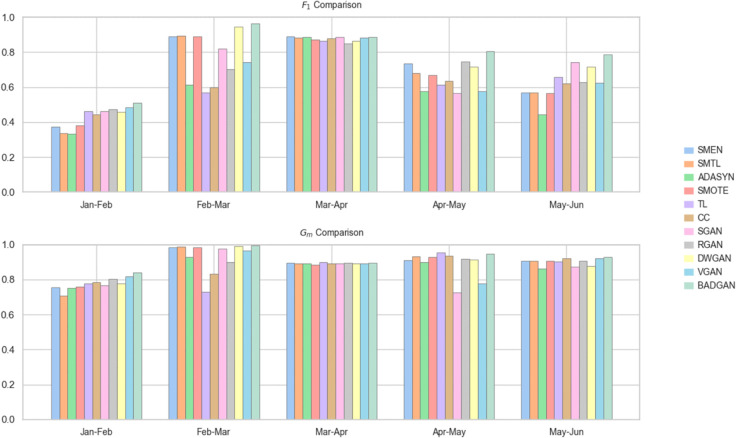
Monthly bar chart with SVC as the classifier.

When using SVC as the classifier, BADGAN demonstrates superior performance across multiple evaluation periods. As shown in Table 3 and Fig 3, it achieves the highest *F*_1_ in four out of five test sets and leads in *G*_*m*_ in three of the test periods. Overall, BADGAN achieves the highest average *F*_1_ and *G*_*m*_, as well as the best *A*_*r*_ across all competitors. It shows an average improvement of 6.76% in *F*_1_ and 3.23% in *G*_*m*_ over the strongest competitors, confirming its ability to maintain a balanced trade-off between sensitivity and specificity in imbalanced FTFD.

**Fig 4:**
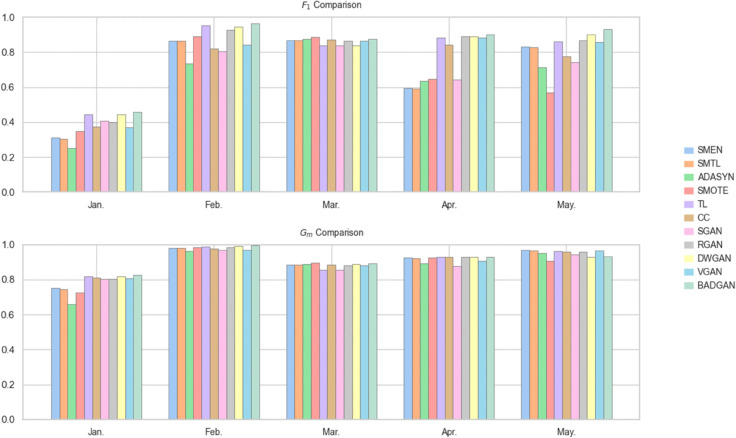
Monthly bar chart with LR as the classifier.

With LR as the classifier, BADGAN continues to exhibit strong performance. As shown in Table 4 and Fig 4, it achieves the highest *F*_1_ and *G*_*m*_ in three out of five evaluation periods. It also records the best average *F*_1_ and *G*_*m*_, securing the top overall ranking among all methods. It outperforms the second-best method by 3.90% in *F*_1_ and 0.30% in *G*_*m*_, demonstrating its robust generalization ability and effectiveness in maintaining a balance between precision and recall across different classification models.

**Fig 5:**
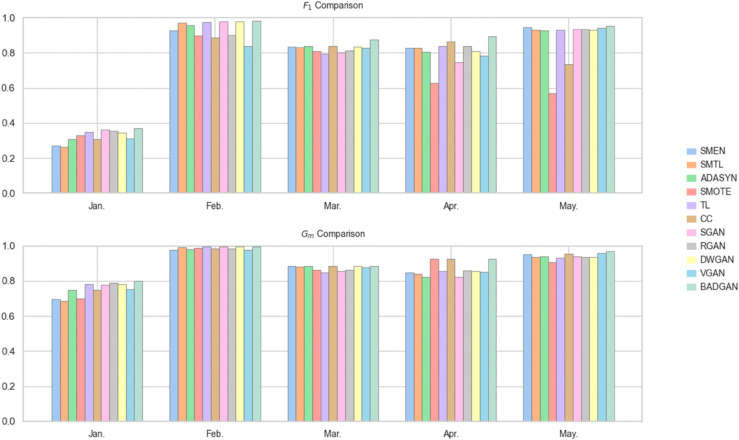
Monthly bar chart with MLP as the classifier.

When using MLP as the classifier, BADGAN demonstrates strong performance in most evaluation periods. As shown in Table 5 and Fig 5, over the five-month evaluation period, it consistently achieves the highest *F*_1_, outperforming the second-best method by an average of 4.46%. In terms of *G*_*m*_, BADGAN leads in three months and remains competitive in the remaining two months, with a performance gap of less than 0.35%. Notably, BADGAN ranks first in both the average *F*_1_ and *G*_*m*_, as well as in *A*_*r*_, confirming its superior capability in capturing both minority and majority class patterns.

In summary, BADGAN consistently outperforms all competing methods across multiple classifiers and evaluation periods. Its superior performance in both *F*_1_ and *G*_*m*_ demonstrates its effectiveness in capturing evolving temporal fraud patterns while maintaining an optimal balance between detecting minority-class fraud and preserving majority-class accuracy. By attaining the highest average performance and top rankings across all evaluated classifiers, BADGAN demonstrates strong generalizability, establishing itself as a reliable and scalable solution for FTFD.

### 5.2 Visualization of varied representations for FTFD

**Fig 6:**
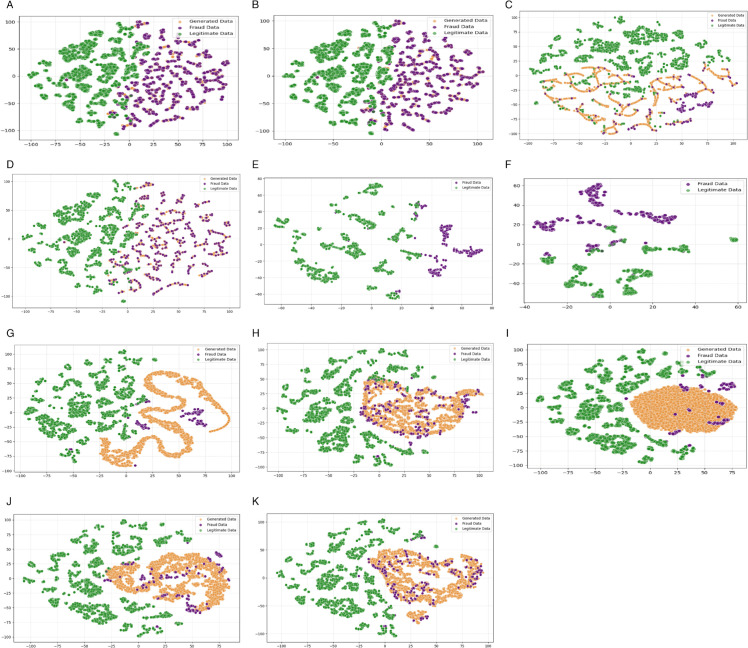
Explorative visualization of varied representations for FTFD. (A) SMEN (B) SMTL (C) ADASYN (D) SMOTE (E) TL (F) CC (G) SGAN (H) RGAN (I) DWGAN (J) VGAN (K) BADGAN.

To evaluate the fidelity of synthetic samples and enhance interpretability, we employ *t*-distributed Stochastic Neighbor Embedding (*t*-SNE) [53] to project high-dimensional transaction data into a two-dimensional space. Fig 6 illustrates the distribution of legitimate, fraudulent, and synthetic samples for all methods, where green, purple, and orange dots represent legitimate, fraudulent, and synthetic samples, respectively.

Hybrid resampling approaches, such as *M*_1_ and *M*_2_ (Fig 6(A)–6(B)), use intelligent synthetic generation techniques to construct locally dense data distributions that preserve the core characteristics of original fraud clusters. By capturing the intrinsic cluster patterns of the minority class, these methods generate synthetic samples that form cohesive structures. However, *M*_1_’s dynamic noise-filtering mechanism and *M*_2_’s adaptive pruning strategy may push certain synthetic instances toward decision-boundary regions. This dispersion effect introduces partial overlap between synthetic and legitimate samples, increasing classifier ambiguity and ultimately degrading precision performance.

Synthetic oversampling techniques, such as *M*_3_ and *M*_4_ (Fig 6(C)–6(D)), generate interpolated minority-class instances to enhance data diversity and distributional continuity. Unlike naive replication methods, these techniques create new samples along feature-space trajectories between existing minority points, thereby improving local density representation. However, their reliance on linear interpolation confines synthetic instances to convex regions within observed minority clusters. This limited extrapolation capability fails to capture the full spectrum of fraudulent patterns, particularly in sparse or non-convex regions, ultimately constraining classifier generalization.

Undersampling methods, such as *M*_5_ and *M*_6_ (Fig 6(E)–6(F)), rebalance class distributions by strategically reducing majority-class samples. *M*_5_ selectively removes borderline instances to sharpen decision boundaries, while *M*_6_ condenses majority clusters by retaining only their centroids. Although both methods effectively mitigate class imbalance, they inherently discard informative majority-class samples, resulting in sparser representations of legitimate transactions. *M*_5_’s boundary-focused pruning may eliminate ambiguous yet potentially valuable instances, while *M*_6_’s centroid-based compression risks losing intra-cluster diversity. Consequently, these methods can weaken the model’s ability to learn robust majority-class patterns, impairing generalization performance.

Generative models, such as *M*_7_–*M*_10_ (Fig 6(G)–6(J)), exhibit distinct patterns in synthetic sample generation relative to traditional resampling methods. *M*_8_ and *M*_10_ generate samples that cluster closely around genuine fraud instances but frequently intrude into legitimate regions, exposing their vulnerability to adversarial imitation and complicating discriminator training. In contrast, *M*_7_ avoids overlap with legitimate areas but suffers from excessive dispersion, failing to align with authentic fraud patterns and inadequately capturing complex fraudulent behaviors. *M*_9_ mitigates these limitations by concentrating synthetic data within compact, well-defined regions, enhancing class separability and reducing boundary intrusion. However, its restrictive generation may lead to high-density clusters that represent only a subset of fraud patterns, increasing the risk of overfitting.

In contrast, *M*_11_ (Fig 6(K)) synthesizes minority-class samples that faithfully adhere to authentic fraud distributions while maintaining robust separation from legitimate instances near decision boundaries. Unlike methods that merely cluster around fraud points, *M*_11_ explicitly optimizes the boundary region by generating high-impact samples in areas where the risk of misclassification is greatest. The resulting synthetic data preserves critical fraud characteristics and reinforces the integrity of the decision boundary, directly enhancing the discriminative model’s training. Compared to conventional approaches, *M*_11_ delivers superior precision in boundary sample generation, which is particularly crucial in severely imbalanced FTFD scenarios.

### 5.3 Parameter sensitivity

To assess the robustness of our approach, we systematically investigate the effect of the nearest-neighbor parameter *k* in Borderline-SMOTE. We test values k∈3,4,5,6,7 and summarize the results in Table 6. These results demonstrate that, as long as *k* remains within an appropriate range, its variation exerts minimal influence on experimental results.

**Table 6:** Sensitivity of the value of *k.*

k	$F_{1}$	$G_{m}$
3	0.8539	0.8889
4	0.8622	0.8902
5	**0.8684**	**0.8974**
6	0.8647	0.8899
7	0.8641	0.8793

Based on these observations, *k* = 5 is selected as the optimal parameter for this study, as it achieves optimal performance in terms of *F*_1_ and *G*_*m*_, while ensuring computational efficiency. This choice optimally balances model accuracy with practical deployment considerations, avoiding noise amplification at lower *k* values and excessive smoothing effects at higher *k* values.

### 5.4 Ablation study

To assess the individual contributions of each component within BADGAN, we conduct an ablation study using a real-world financial transaction dataset. The comparative results are summarized in Table 7. We evaluate three model variants: *A*_0_ represents the baseline GAN, *A*_1_ represents the addition of only the boundary sample discriminator on top of *A*_0_, and *A*_2_ corresponds to the full implementation of our proposed model, BADGAN, which incorporates distance-adversarial learning. SVC is employed as the downstream classifier for all evaluations to ensure consistency.

**Table 7:** Ablation study.

Train	Test	Criteria	$A_{0}$	$A_{1}$	$A_{2}$
Jan.	Feb.	*F*_1_	0.3312	**0.4645**	0.4594
		*G*_*m*_	0.7562	**0.7839**	0.7760
Feb.	Mar.	*F*_1_	0.7881	0.9377	**0.9909**
		*G*_*m*_	0.9727	0.9912	**0.9967**
Mar.	Apr.	*F*_1_	**0.8863**	0.8876	0.8671
		*G*_*m*_	0.8931	0.8932	**0.8940**
Apr.	May.	*F*_1_	0.5331	0.5438	**0.6534**
		*G*_*m*_	0.7354	0.7270	**0.7648**
May.	Jun.	*F*_1_	0.5941	0.7552	**0.7845**
		*G*_*m*_	0.9060	0.8783	**0.9392**
$A_{v}$		*F*_1_	0.6266	0.7178	**0.7511**
		*G*_*m*_	0.8527	0.8547	**0.8741**
*A*_*r*_		*F*_1_	2.8000	**1.6000**	**1.6000**
		*G*_*m*_	2.6000	2.2000	**1.2000**

As shown in Table 7, both *A*_1_ and *A*_2_ substantially outperform *A*_0_ in terms of *F*_1_ and *G*_*m*_. This indicates that enhancing the discriminative power of boundary samples is a key factor in improving the quality of the generated data. The improved performance of *A*_2_ over *A*_1_ further confirms the effectiveness of distance-adversarial learning. BADGAN enhances the discriminative power of generated samples through the boundary sample discriminator and constrains their distribution authenticity through distance-adversarial learning, significantly improving the recall and accuracy of downstream fraud detection.

These findings underscore the pivotal role of boundary-aware mechanisms in FTFD generation modeling. The proposed architectural innovation not only significantly enhances the model’s capacity to represent complex fraud patterns but also improves its adaptability and generalization in adversarial environments, where fraudsters deliberately disguise their behavior to evade detection. This advancement offers a novel technological pathway for applying generative adversarial networks in financial risk control, demonstrating particularly strong effectiveness in addressing highly imbalanced fraud detection tasks.

### 5.5 Experiments on imbalanced classification datasets

To further evaluate the generalizability and effectiveness of BADGAN beyond the financial domain, we conduct a comprehensive benchmark study on three widely used imbalanced classification datasets from the UCI (https://archive.ics.uci.edu/ml/index.php) (Dataset *D*_1_: Default of credit card clients), Synthetic Financial Datasets For Fraud Detection (https://www.kaggle.com/datasets/ealaxi/paysim1) (Dataset *D*_2_: Paysim), and Credit Card Fraud Detection (https://www.kaggle.com/datasets/mlg-ulb/creditcardfraud) (Dataset *D*_3_: Creditcard) repository [54]. For brevity, we refer to these datasets as *D*_1_, *D*_2_, and *D*_3_, respectively. Table 8 provides descriptions of the different datasets, where #S and #A represent the number of samples and attributes, respectively. #IR indicates the imbalance ratio. This evaluation aims to assess the model’s adaptability across diverse data distributions. To ensure fair and consistent comparisons across all methods, we use SVC as the base classifier.

**Table 8:** Public datasets.

Abbr.	#S	#A	#LR
*D*_1_	30000	24	0.2212
*D*_2_	1048575	10	0.0010
*D*_3_	284807	31	0.0017

As shown in Table 9, BADGAN consistently outperforms all ten compared baseline methods. It consistently achieves the highest *F*_1_ across all datasets and delivers the best overall performance in terms of *G*_*m*_, with only a minor exception of scoring 0.71% lower than ADASYN on one dataset. Importantly, BADGAN ranks first in both the average metric values and average rankings for *F*_1_ and *G*_*m*_. These results highlight the model’s effectiveness in addressing class imbalance while maintaining strong generalization across diverse scenarios. Furthermore, its robustness against adversarial evasion demonstrates its suitability for real-world applications with dynamic data distributions.

**Table 9:** Imbalanced classification performance of eleven methods.

Dataset	Criteria	$M_{1}$	$M_{2}$	$M_{3}$	$M_{4}$	$M_{5}$	$M_{6}$	$M_{7}$	$M_{8}$	$M_{9}$	$M_{10}$	$M_{11}$
*D*_1_	*F*_1_	0.1854	0.1865	0.1864	0.1865	0.1847	0.1753	0.1216	0.1126	0.1718	0.1718	**0.2043**
	*G*_*m*_	0.5471	0.5610	0.5577	0.5597	0.5579	0.4760	0.4445	0.4065	0.5137	0.5137	**0.5674**
*D*_2_	*F*_1_	0.8468	0.8633	0.7891	0.8641	0.9052	0.8478	0.9084	0.9116	0.8712	0.8712	**0.9166**
	*G*_*m*_	0.9202	0.9470	**0.9578**	0.9501	0.9200	0.9208	0.9203	0.9318	0.9206	0.9263	0.9510
*D*_3_	*F*_1_	0.5480	0.6487	0.4987	0.6324	0.6433	0.5594	0.4888	0.5628	0.6588	0.6588	**0.6708**
	*G*_*m*_	0.8060	0.8112	0.8177	0.8090	0.6902	0.6541	0.7539	0.6561	0.7964	0.7964	**0.8212**
$A_{v}$	*F*_1_	0.5267	0.5662	0.4914	0.5610	0.5777	0.5275	0.5063	0.5290	0.5673	0.5673	**0.5972**
	*G*_*m*_	0.7578	0.7731	0.7777	0.7729	0.7227	0.6836	0.7062	0.6611	0.7455	0.7455	**0.7799**
*A*_*r*_	*F*_1_	7.6667	4.0000	8.3333	4.3333	4.6667	7.6667	8.0000	6.6667	5.0000	5.0000	**1.0000**
	*G*_*m*_	7.0000	3.0000	2.6667	3.3333	8.0000	9.0000	9.0000	9.6667	6.6667	6.6667	**1.3333**

### 5.6 Discussion

The proposed BADGAN introduces a novel approach to financial fraud detection by integrating a dual-discriminator architecture with Borderline-SMOTE to enhance boundary-sample learning. While effective in generating high-quality synthetic samples and addressing class imbalance, several limitations require consideration. The computational overhead associated with the dual-discriminator design may impede real-time deployment, highlighting the need for more efficient architectures, such as parameter-sharing discriminators or lightweight temporal encoders. Additionally, the current model fails to explicitly capture the temporal dependencies in transaction sequences, which limits its capability to model evolving fraud patterns. To address this issue, future research directions will include the integration of Recurrent Neural Networks (RNNs) [55] and attention mechanism-based modules for the explicit capture of temporal dependencies in transaction sequences. These methods can better track the evolution of fraud patterns, thereby improving the model’s adaptability and accuracy in dynamic financial environments.

A key limitation of the current framework is its inability to capture the relational and spatial dimensions of fraud, such as coordinated attacks across multiple accounts or geographically clustered activities. Future work could investigate graph-based approaches to model inter-account relationships and spatial attention mechanisms for location-aware detection. Furthermore, the framework’s performance may deteriorate under extreme class imbalance, where the scarcity of genuine fraud samples limits the quality of generated synthetic samples. Incorporating semi-supervised learning techniques or adaptive resampling strategies offers promising directions to address this challenge.

Future research will focus on improving the interpretability of BADGAN and enhancing its transparency in practical applications. We plan to introduce attention-based visualization techniques to help analysts intuitively understand the key factors in the model’s decision-making process. Meanwhile, counterfactual explanation methods will be applied to reveal the minimal input changes that affect the model outputs, providing actionable insights for fraud analysts. In addition, we will evaluate the model’s performance in a streaming data environment to further verify its robustness in addressing evolving patterns and methods of financial fraud.

Recent studies show that adversarial attacks pose significant risks to machine learning models, particularly in sensitive domains such as fraud detection [56]. Although BADGAN demonstrates strong performance across varying fraud patterns, the present work does not examine its behavior under adversarial manipulation. We recognize this as an important direction for future research and consider adversarial robustness evaluation, such as evasion and data-poisoning scenarios, a valuable extension of our framework.

Overall, BADGAN shows strong potential in tackling critical challenges in fraud detection, particularly in handling class imbalance and generating high-quality synthetic samples. However, to enhance its real-world applicability, future research should focus on improving its computational efficiency, capturing temporal dependencies in transaction sequences, and incorporating relational awareness for detecting coordinated fraud activities. Additionally, while the model performs well under typical conditions, its robustness to adversarial attacks has not been explicitly tested. Addressing this gap will be crucial, as adversarial threats pose a growing concern in machine learning, particularly in sensitive applications like fraud detection. Future work should explore strategies to enhance the model’s resilience against evasion and poisoning attacks. Finally, integrating BADGAN’s boundary-sensitive design with temporal modeling techniques could offer more adaptive and comprehensive fraud detection solutions in dynamic financial environments.

## 6 Conclusion

This study presents BADGAN, a novel boundary-aware dual-discriminator GAN framework designed to address class imbalance in financial fraud detection. Unlike conventional oversampling methods, BADGAN employs a boundary-aware generation strategy to synthesize high-quality minority-class samples that reinforce decision boundaries while preserving distributional authenticity. By integrating adversarial distance constraints with a dual-discriminator architecture, the model achieves a balance between sample diversity and discriminative strength, enabling fraud detectors to capture subtle anomalous patterns more effectively. Extensive evaluations on real-world transaction data and public benchmarks demonstrate BADGAN’s consistent superiority over state-of-the-art methods, particularly under highly imbalanced and evolving fraud scenarios. The boundary-aware design not only improves classifier robustness but also mitigates synthetic sample overfitting—a common drawback of GAN-based approaches. Future work will focus on dynamic boundary adaptation to accommodate non-stationary fraud behaviors and extensions to cross-modal financial data, such as graph-structured transactions. Additionally, incorporating self-supervised pre-training may further reduce reliance on labeled data. Beyond fraud detection, BADGAN’s adaptability highlights its potential in broader domains, such as cybersecurity and medical anomaly detection, enabling more resilient AI-driven risk management.
